# Local surface plasmon enhanced polarization and internal quantum efficiency of deep ultraviolet emissions from AlGaN-based quantum wells

**DOI:** 10.1038/s41598-017-02590-7

**Published:** 2017-05-24

**Authors:** Cai Zhang, Ning Tang, Liangliang Shang, Lei Fu, Weiying Wang, Fujun Xu, Xinqiang Wang, Weikun Ge, Bo Shen

**Affiliations:** 1 State Key Laboratory of Artificial Microstructure and Mesoscopic Physics, School of Physics, Peking University, Beijing, 100871 China; 2 Collaborative Innovation Center of Quantum Matter, Beijing, 100871 China

## Abstract

We report the enhancement of the polarization and internal quantum efficiency (IQE) of deep-UV LEDs by evaporating Al nanoparticles on the device surface to induce localized surface plasmons (LSPs). The deep-UV LEDs polarization is improved due to part of TM emission turns into TE emission through LSPs coupling. The significantly enhanced IQE is attributed to LSPs coupling, which suppress the participation of delocalized and dissociated excitons to non-radiative recombination process.

## Introduction

In the past decade, AlGaN-based deep UV LEDs have attracted considerable attentions owing to their great application potential in biological detection and optical catalysis. Luminescence from 210 nm to 365 nm have been obtained by modulating the Al compositions in the Al_x_Ga_1-x_N active layer^1^. However, deep UV-LEDs still suffer from relatively low external quantum efficiency (EQE) in contrast to commercial blue LEDs^2^. It is well-known that EQE depends on internal quantum efficiency (IQE) and light extraction efficiency (LEE)^3^. High density of intrinsically produced nonradiative impurities and dislocations during the growth of AlGaN materials are the major limitations for achieving high IQE^4, 5^. And only the TE mode generated in the active layers can escape from the top and bottom surfaces when it is inside an escape cone. However, the TM mode is the favored polarization of the luminescence from high Al composition Al_x_Ga_1−x_N alloys. It is thus an obstacle for achieving high LEE^6^. Many works focus on improving material quality to achieve higher IQE and tuning the valence subbands in order to enhance the TE polarized emission^7, 8^. However, the performance of deep UV-LEDs have yet been satisfied with these traditional approaches.

Among many recent attempts, some researchers take advantage of localized surface plasmons (LSPs) to enhance the IQE of UV LEDs. One main obstacle for SP-enhanced AlGaN-based deep UV LEDs is to achieve effective and energy-matched coupling. However, the SP energy of Ag and Au on UV LEDs are approximately 2.76 eV (450 nm) and 2.2 eV (560 nm)^9^. The SP energy of Al on UV LEDs is higher than 5 eV (250 nm). The real part of the dielectric constant is negative over the whole wavelength region for UV light. It provides a broad resonance spectrum, being the best choice for deep UV LEDs, which can easily match the momentum of all photons generated in the active layer of LEDs^10^.

In this letter, a 10-nm-thick Al film is deposited on the top of AlGaN multiple quantum wells (MQWs), and an annealing process is then used to fabricate Al nanoparticles (NPs). LSPs are generated at the interface between the AlGaN cap layer and Al NPs. The in-plane (TE) polarization is improved from 13.7% to 19.7%. A 3D finite-difference-time-domain (FDTD) simulation is used to calculate the Purcell enhancement factors for TE and TM modes. Temperature dependent photoluminescence (PL) intensities and peak positions are investigated for understanding the mechanism of LSPs coupling. It is found that the IQE is enhanced due to the coupling of LSPs with delocalized and dissociated excitons. We believe that the enhanced polarization is attributed to the LSPs coupling process, where part of TM emission turns into TE emission.

**Figure 1:**
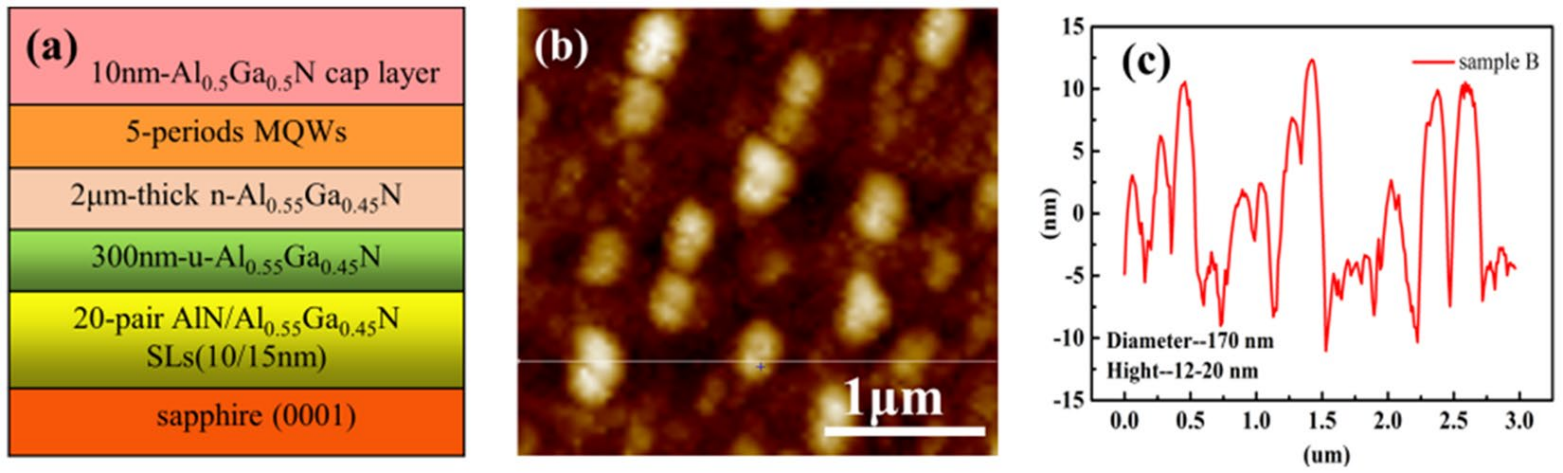
(**a**) Schematic illustration of the structure for sample A; (**b**) Atomic force microscopy (AFM) topographic image of Al particles on AlGaN surface of sample B, which are formed by annealing 30 mins in N_2_; (**c**) The AFM image of the depth profile along the dashed line in Fig. 1(b).

For the sake of comparison, two samples were used in the experiments, one (sample B) with LSPs, and the other (sample A) without. The structure of sample A is shown in Fig. 1(a), the sample B is a similar MQWs but with LSPs. A 10 nm thin layer of Al is deposited on MQWs using e-beam evaporation, we have used a vacuum packing to protect the Al film from oxidation. Then, it followed by a thermal annealing process at 550°for 30 min in N_2_ atmosphere to form self-assembled particles. Figure 1(b) and (c) show atomic force microscopy (AFM) images of Al NPs after annealing. The diameter and the height of the Al NPs are 170 nm and 12–20 nm, respectively.

**Figure 2:**
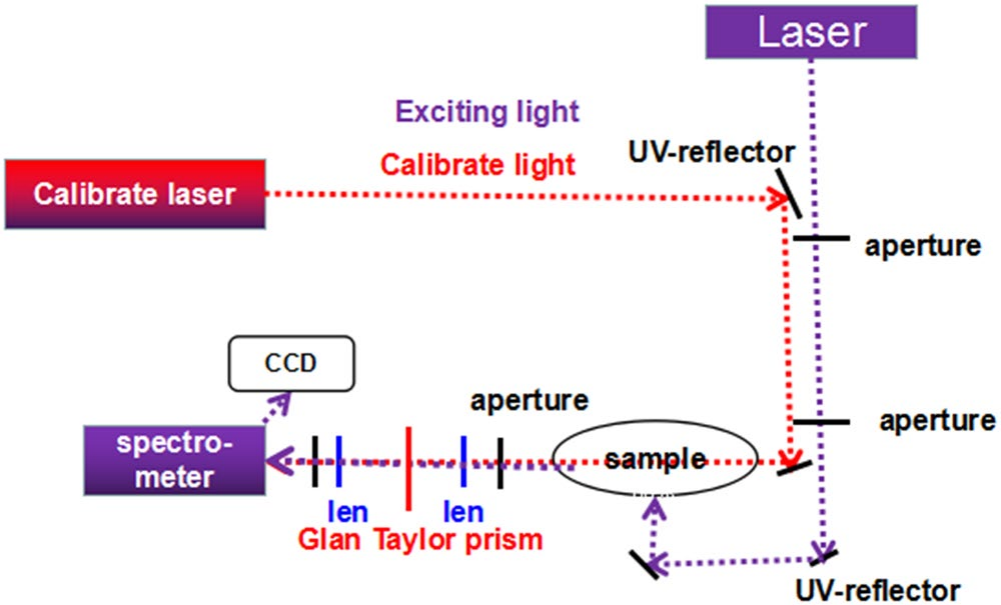
The polarization photoluminescence experimental light path diagram.

For the polarization PL, the experimental apparatus includes as shown in Fig. 2: 266 nm neodymium-doped yttrium aluminum garnet laser (YAG: Nd laser), Glan Taylor prism, spectrometer, CCD camera, lenses and other optical components. The laser hits on the c-face of sample, the the signal is collected and analyzed by a Glan-Taylor prism from side of sample. The entire setup was carefully calibrated using an unpolarized light source with a second polarizer. For the temperature dependent PL spectra measurement, the laser hits on the c-face and the signal is also collected from the c-face.

**Figure 3:**
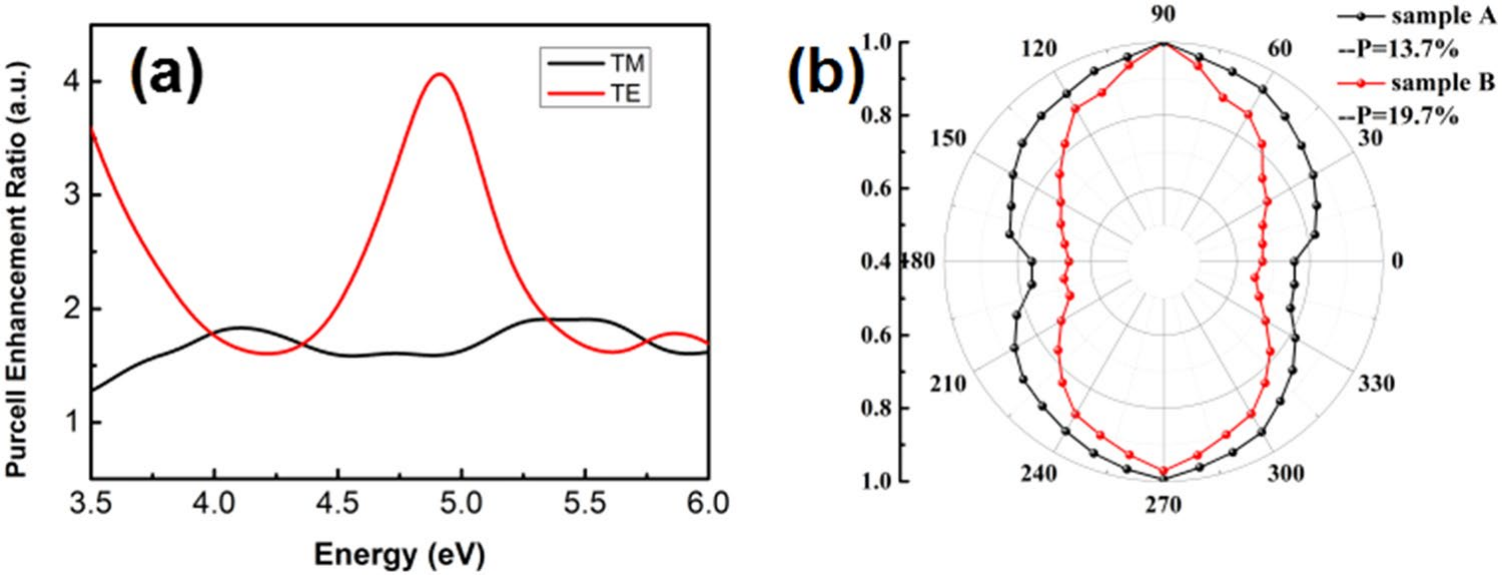
(**a**) The PL spectrum consists of TE mode and TM mode for sample A (black) and sample B (red); (**b**) Normalized polarization diagram of sample A (black) and sample B (red).

Figure 3(a) shows the intensities of the TE and TM modes for the two samples at 300 K. The intensities of the two modes are both enhanced for sample B, in comparison to sample A. It is found that the enhancement ratio of TE modes is larger than that of TM modes. The in-plane polarization (P) of a (0001) oriented device is defined by^11^:

1 $${\rm P}=\frac{{I}_{TE}-{I}_{TM}}{{I}_{TE}+{I}_{TM}},$$

where *I*_*TE*_ and *I*_*TM*_ are the integrated TE-polarized and TM-polarized light intensities, respectively. Figure 3(b) shows the P values for sample A (black) and B (red), respectively. The polarized angle of 0 degree is defined as TM mode, while 90 degree is TE mode. One can see that P increases from 13.7% for sample A to 19.7% for sample B. The different enhancement ratios and increased P value indicate the occurrence of LSP-MQWs coupling and the improvement of LEE.

**Figure 4:**
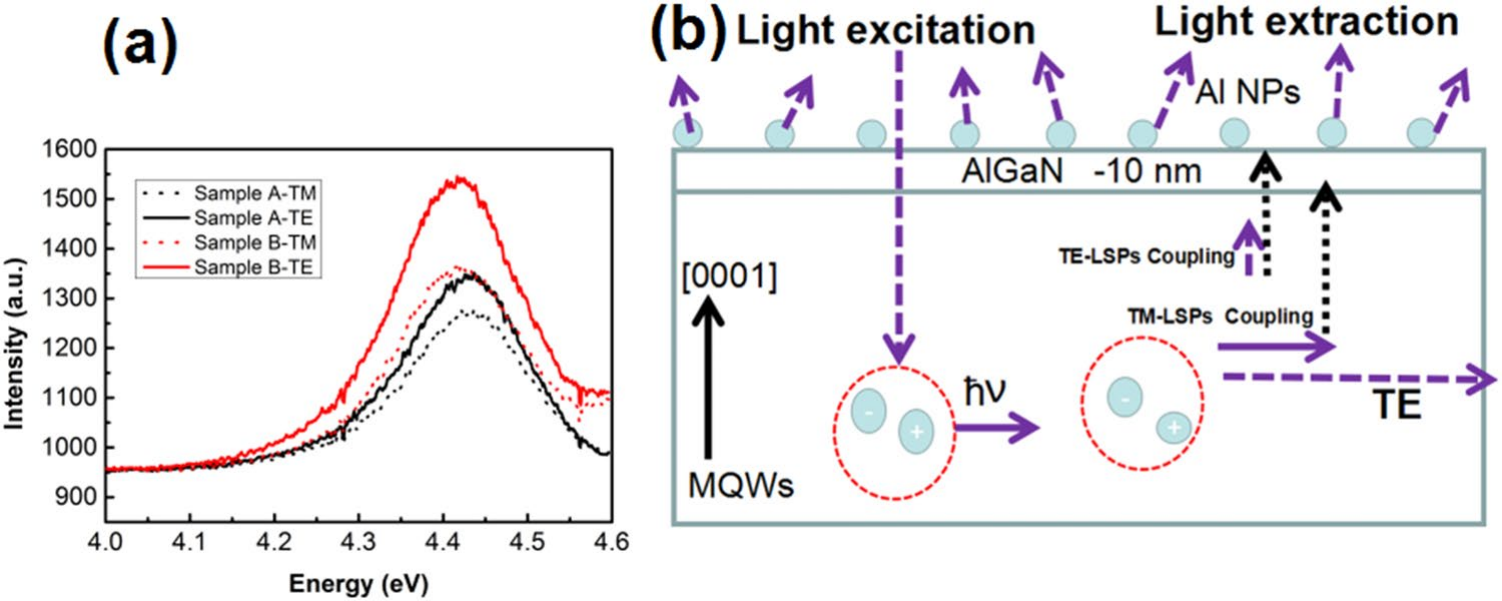
(**a**) Wavelength dependent Purcell enhancement ratios of TE mode and TM mode, which are calculated to describe the enhancement ratio of the radiative recombination rate; (**b**) A schematic illustration of the LSPs enhanced deep UV MQWs in this study.

To explore the LSPs-MQWs coupling behavior, 3D FDTD simulation is carried out, as shown in Fig. 4. An Al circle (diameter = 170 nm, height = 16 nm) is placed on the top of the Al_0.5_Ga_0.5_N (n = 2.4) cap layer^12^. The perfectly matched layer (PML) absorbing boundaries are assumed on all sides, and the FDTD simulation domain is 2 μm *2 μm *1.6 μm (x, y, and z directions). The initial settings use a mesh size of *dx* = *2 *nm for a dipole-circle distance of 10 nm. Here the TE and TM mode dipoles are considered. The Purcell enhancement factor *F*_*p*_ *(w)* quantifies the increase of the radiative recombination rate, which can be described as^13^:

2 $${F}_{P}(\omega )=\frac{{P}^{\ast }}{{P}_{0}}=\frac{{k}^{\ast }(w)}{k(w)}.$$

Where *k* ^*^ *(w)* is the radiative recombination rate with LSPs coupling, the *k(w)* is that without LSPs coupling. According to the assumption by FDTD, where *P*_*0*_ is the radiation power of a classical dipole in a homogeneous background, which in our case is air/vacuum. *P* ^*^ is the radiation power of the dipole in the proximity of the metal NPs. The radiation power recorded is thus corresponding to the radiative recombination rate^14^. It is found that the radiative recombination rates at 280 nm are increased about 3.5 times for the TE mode while 1.7 times for the TM mode, which qualitatively agrees with the experimental data above.

The schematic illustration of LSPs enhanced deep UV MQWs is shown in Fig. 4(b). The LSPs couple with either TE mode emission or TM mode emission, and then emit light through Al NPs efficiently. As such, part of TM emission turns into TE emission, which results in a higher enhancement ratio for TE mode. The polarization is thus enhanced by LSPs coupling^15^. Besides, the radiative recombination rates enhancement should certainly promote the IQE.

**Figure 5:**
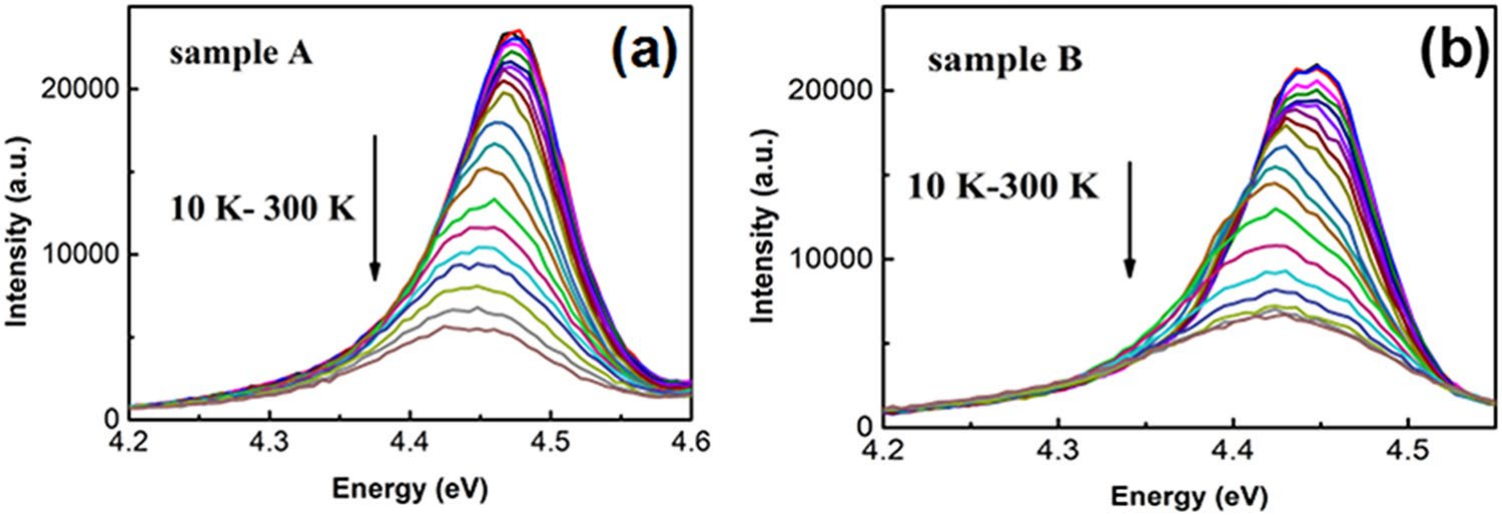
(**a**) Temperature dependent PL spectrum of sample A; (**b**) Temperature dependent PL spectrum of sample B.

Figure 5 are the temperature dependent PL spectra of sample A (a) and sample B (b), respectively. The luminescence peaks are close to 280 nm (4.43 eV). In order to get the values of IQE, the PL intensities of each sample are normalized to the intensity at 10 K. In general, the IQE of semiconductor sample is defined as the ratio of the integrated PL intensity at 300 K over that at 10 K. It is found that the IQE of sample B (53.5%) is much higher than that of sample A (32.7%), as shown in Fig. 5(b).

**Figure 6:**
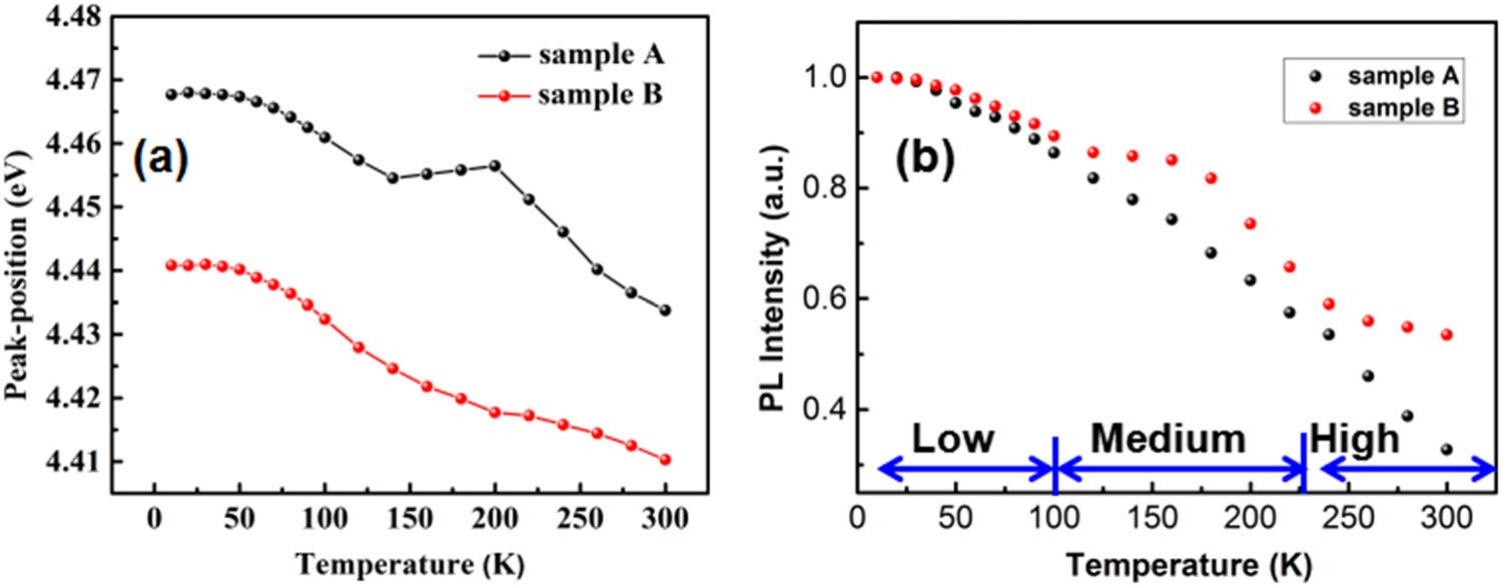
(**a**) Temperature dependent PL peak positions of the sample A (black) and sample B (red); (**b**) Temperature dependent integrated PL intensities of the sample A (black) and sample B (red).

For understanding the LSPs coupling features, the temperature dependent peak energies are shown in Fig. 6(a). One can see an S-shape variation of the peak energy with temperature for sample A, while there is no such feature for sample B. The S-shape PL spectrum is a strong evidence for identifying the carrier localization behavior^16^. In the low temperature region, the carriers have little thermal energy to overcome the localization barrier. The PL peak energies and intensities of two samples have almost no difference as shown in Fig. 5(b). While in the medium temperature range, the localized carriers start to escape from the potential well to become free. The blue shift of the peak energy for sample A is just caused by the delocalization of the carriers. On the contrary, the LSPs of sample B as an ultra-fast recombination channel couple with the delocalized free carriers to emit light. The additional contribution of the LSPs coupled radiative recombination results in the observation of a red shift, as shown in Fig. 6(a). Moreover, the intensity quenching with increasing temperature is slower for sample B, as shown in Fig. 6(b), leading to a higher PL intensity from sample B than that from sample A. There is a similar trend in the high-temperature range, where LSPs effectively couple with dissociated free carriers. It can be inferred from the analysis above that the LSPs coupling process quickly empties the localized and bound excitons and speeds up the radiative transition process. Due to the fast nature of the LSP–exciton coupling process, the radiative recombination would be enhanced, and thus the total emission efficiency can be enhanced.

In summary, we report that by LSP coupling the PL polarization from Al_0.37_Ga_0.63_N/Al_0.5_Ga_0.5_N MQWs is enhanced by about 43%. The mechanism for that enhancement is attributed to the LSPs-QW coupling, which results in part of TM emission turns into TE emission. 3D FDTD simulation is implemented to calculate the Purcell enhancement ratios of the sample (B) with LSPs, which illustrates that the radiative recombination rate of the TE mode achieves a more remarkable improvement. The significant IQE enhancement, as high as 63%, was also achieved for AlGaN-based MQWs by LSPs coupling. The improvement of the IQE originates from an increased radiative recombination rate by LSPs coupling with delocalized and dissociated excitons.

## References

[1] He, C. *et al*. Free and bound excitonic effects in Al0.5Ga0.5 N/Al0.35Ga0.65 N MQWs with different Si-doping levels in the well layers. *Sci Rep*. **5** (2015). 10.1038/srep13046 PMC4533523 26267249

[2] Shatalov M (2012). AlGaN Deep-Ultraviolet Light-Emitting Diodes with External Quantum Efficiency above 10%. Appl. Phys. Express.

[3] Hirayama H (2007). 231–261 nm AlGaN deep-ultraviolet light-emitting diodes fabricated on AlN multilayer buffers grown by ammonia pulse-flow method on sapphire. Appl. Phys. Lett..

[4] Deguchi T (1999). Quantum-Confined Stark Effect in an AlGaN/GaN/AlGaN Single Quantum Well. Structure. Jpn. J. Appl. Phys.

[5] Reich C (2013). Excitonic recombination in epitaxial lateral overgrown AlN on sapphire. Appl. Phys. Lett..

[6] Oder TN (2004). III-nitride blue and ultraviolet photonic crystal light emitting diodes. Appl. Phys. Lett..

[7] Al Tahtamouni TM, Lin JY, Jiang HX (2012). Optical polarization in c-plane Al-rich AlN/AlxGa1-xN single quantum wells. Appl. Phys. Lett..

[8] Kolbe T (2010). Optical polarization characteristics of ultraviolet (In)(Al)GaN multiple quantum well light emitting diodes. Appl. Phys. Lett..

[9] Okamoto K (2004). Surface-plasmon-enhanced light emitters based on InGaN quantum well. Nat. Mater..

[10] Jiang S (2015). The Coupling Behavior of Multiple Dipoles and Localized Surface Plasmons in Ag Nanoparticles Array. Plasmonics.

[11] Reich C (2015). Strongly transverse-electric-polarized emission from deep ultraviolet AlGaN quantum well light emitting diodes. Appl. Phys. Lett..

[12] Huang K (2014). Top- and bottom-emission-enhanced electroluminescence of deep-UV light-emitting diodes induced by localised surface plasmons. Sci. Rep..

[13] Purcell, E. M. Spontaneous Emission Probabilities at Radio Frequencies (Springer, 1995).

[14] Kaminski F, Sandoghdar V, Agio M (2007). Finite-Difference Time-Domain Modeling of Decay Rates in the Near Field of Metal Nanostructures. J. Comput. Theor. Nanosci..

[15] Gao N (2012). Surface-plasmon-enhanced deep-UV light emitting diodes based on AlGaN multi-quantum wells. Sci. Rep..

[16] Li J (2003). Band structure and fundamental optical transitions in wurtzite AlN. Appl. Phys. Lett..

